# Direct Defluorination and Amination of Polytetrafluoroethylene and Other Fluoropolymers by Lithium Alkylamides

**DOI:** 10.3390/molecules29133045

**Published:** 2024-06-26

**Authors:** Guillaume Herlem, Yaelle Roina, Mathieu Fregnaux, Anne-Marie Gonçalves, Hélène Cattey, Fabien Picaud, Frédéric Auber

**Affiliations:** 1 Laboratoire SINERGIES, CHU Jean Minjoz, UFR Sciences & Techniques, 16 Route de Gray, 25030 Besançon CEDEX, France; 2 Institut Lavoisier de Versailles, Université Paris-Saclay, UVSQ, CNRS, UMR 8180, 78035 Versailles CEDEX, France; 3 Institut ICMUB—CNRS 6302, Université de Bourgogne, UFR Sciences et Techniques Mirande, 9 Avenue Alain Savary, 21000 Dijon CEDEX, France

**Keywords:** PTFE, Nafion, PVDF, fluoropolymers, amination, defluorination

## Abstract

Polytetrafluoroethylene (PTFE) and, by extension, fluoropolymers are ubiquitous in science, life, and the environment as perfluoroalkyl pollutants (PFAS). In all cases, it is difficult to transform these materials due to their chemical inertness. Herein, we report a direct amination process of PTFE and some fluoropolymers such as polyvinylidene fluoride (PVDF) and Nafion by lithium alkylamide salts. Synthesizing these reactants extemporaneously between lithium metal and an aliphatic primary di- or triamine that also serves as a solvent leads to the rapid nucleophilic substitution of fluoride by an alkylamide moiety when in contact with the fluoropolymer. Moreover, lithium alkylamides dissolved in suitable solvents other than amines can react with fluoropolymers. This highly efficient one-pot process opens the way for further surface or bulk modification if needed, providing an easy, inexpensive, and fast experiment protocol on large scales.

## 1. Introduction

PTFE and, by extension, fluoropolymers are some of the most important polymer materials that have a significant impact on our daily lives, from food (antiadhesive pan) to rechargeable batteries (lithium battery separators) as well as health (prostheses and drugs). They have many desirable properties such as inertness to chemicals, temperature, and aging, mainly. However, the stable C-F bond (485 kJ/mol) and low surface energy towards oils and many solvents including water make them challenging to functionalize, limiting some of their applications [1,2,3].

The modification of fluoropolymers, whether on the surface to add a functional group or bulk until their depolymerization, requires their defluorination. Several solutions exist, such as wet or plasma etching or mineralization [4,5,6,7,8]. Originally, it was performed by dissolving lithium or sodium in liquid ammonia, following the Billups–Birch reaction [9,10]. Then, another alternative method was to use sodium naphthalenide dissolved in an ether such as tetrahydrofuran (THF) to reduce health risks or benzoin in dimethylsulphoxide [11,12]. More recently, a new defluorination possibility was proposed with a magnesium salt in a mixture of benzene and 4-(dimethylamino)pyridine [13].

Despite many works reporting the functionalization or depolymerization of fluoropolymers [14], there is no direct chemical conversion of carbon–fluorine bonds in fluorinated materials to any functionality [15]. Alkali metals are known to be soluble in light amines to a lesser extent than in ammonia, while ethylenediamine (EDA) improves the solubility of alkali metals in light amines. Extensive work with lithium in light amines or a mixture of light amines and EDA (in a small amount) has been dedicated to the reduction in several types of aromatic compounds. Especially, the Benkeser reduction, which is a fork of the Billups–Birch reaction [16], overcomes the drawbacks of handling ammonia when performed on kilograms or even an industrial scale [17]. But there is no mention of the corresponding alkali alkylamide salt formation in EDA or an explanation of its reactivity [18,19,20,21,22,23]. More recently, the alkylation of single-walled carbon nanotubes by lithium dissolved in EDA has been reported [24,25,26], while reduced graphene oxide is aminated [27].

Here, we report a successful approach towards an experimental one-pot defluorination and amination of fluoropolymers. A stable but fluoropolymer-reactive lithium alkylamide salt is generated in situ, in mild conditions (20 °C) and under an inert atmosphere (argon), by the reaction between lithium and a primary di- or triamine. EDA, diethylenetriamine (DETA), and 1,3-diaminopropane (DAP) are three solvents that react with lithium by forming stable dark blue solvated electrons until the end of the reaction, leading to the alkylamide formation and hydrogen bubbling. Herein, PTFE, polyvinylidene fluoride (PVDF), and a sulfonated tetrafluoroethylene-based fluoropolymer-copolymer (Nafion) were reacted with lithium alkylamides as a proof of concept.

## 2. Results and Discussion

### 2.1. Synthesis of Lithium Alkylamides in Aliphatic Primary Di- or Triamines

Organolithium compounds are of great importance in organic asymmetric synthesis [28,29] but remain little studied in the case of lithium alkylamides because few crystallographic data exist. They are more soluble and reactive than their sodium or potassium counterparts, hence the use of lithium in our study [30,31].

**Figure 1:**
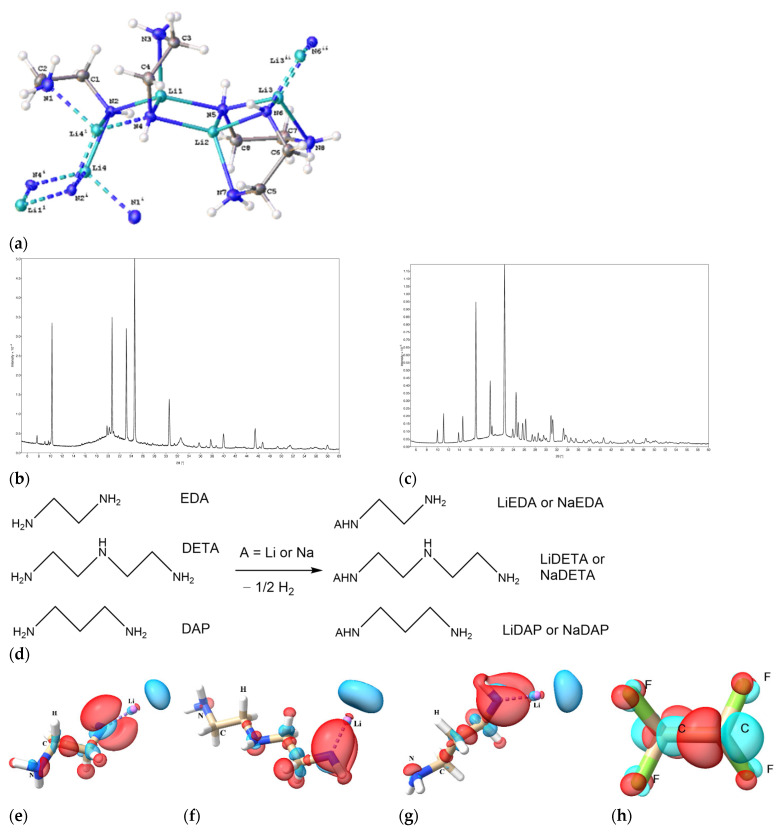
Synthesis of lithium alkylamides, their reactive centers, and the crystal structure of LiEDA. The XRD structure of (**a**) LiEDA synthesized from the fast reaction between Li and EDA in stoichiometric proportions. The powder XRD of (**b**) LiDETA and (**c**) LiDAP. (**d**) The synthesis scheme of LiEDA, LiDETA, and LiDAP by the reaction of lithium with EDA, DETA, or DAP, respectively. Isosurface maps of dual descriptors (Δ*f*) for (**e**) LiEDA, (**f**) LiDETA, (**g**) LiDAP, and (**h**) PTFE. Positive and negative regions of the dual descriptor Δ*f* are represented as blue and red colors, respectively.

In stoichiometric proportions between lithium and EDA, DETA, or DAP, all compounds are consumed until a white salt is formed. The X-ray diffraction (XRD) analysis of the crystals obtained by the reaction between lithium and EDA (Figure 1a) leads to the chemical formulae C_8_H_28_Li_4_N_8_ in the unit cell (space group P2_1_/n) and corresponds to LiNHC_2_H_4_NH_2_ (LiEDA), in total agreement with the only known structure of a lithium alkylamide obtained by the reaction of Li_3_N with EDA for 3 days, instead of less than 1 h in our cases [32]. So far, Beumel et al. obtained only a white suspension, by an equimolar synthesis between lithium and EDA, but without achieving a total reaction of a completely solid lithium amide [33]. The powder diffractograms of the total reaction between lithium and DETA (Figure 1b) and LiDAP (Figure 1c) show diffraction peaks characteristic of crystalline LiDETA and LiDAP, respectively. A lithium atom replaces a hydrogen atom on one of the two primary amino groups. We formalized the general reaction equation in Figure 1d.

When the lithium metal is below stoichiometry relative to EDA, DETA, or DAP, the resulting alkylamide salt remains in solution in the excess solvent. Immersed in this reaction medium, the fluoropolymers (PTFE, PVDF, and Nafion) quickly take on a black color.

### 2.2. Reactivity of Lithium Alkylamides towards the C-F Bond in PTFE

As expected and confirmed theoretically by the dual descriptor isosurfaces related to lithium alkylamides (Figure 1e–h) within the framework of conceptual density functional theory (CDFT) [34,35,36], the alkylamide group (bearing the biggest red lobe) is a favorable site for an electrophilic attack (Table 1).

**Scheme 1:**
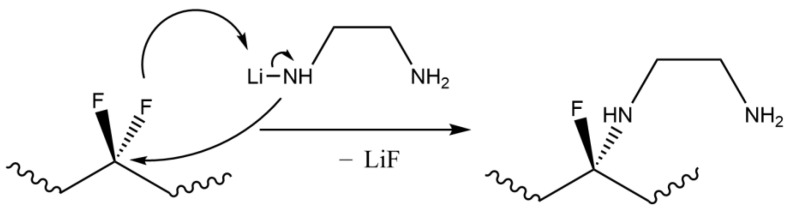
Suggested reaction mechanism between a fluoropolymer and the lithium alkylamide.

On the contrary, lithium (blue lobe) is a favorable site for nucleophilic attacks. Indeed, the local reactivity descriptors *f*^+^, *f*^−^, and *f^o^* (Fukui indices) related to nucleophilic, electrophilic, and radical attacks can be gathered via the condensed dual descriptor Δ*f* to reveal reactive sites at a glance [37,38]. However, the biggest contribution of the blue isolated lobe in front of the lithium atom comes from *f*^+^, but the contribution of *f^o^* is not negligeable at all (no contribution from *f*^−^). Lithium alkylamides are very reactive towards PTFE, as shown by local descriptors, but are highly dependent on their aggregation in a solution [39,40,41,42,43,44]. In all cases (LiEDA, LiDETA, and LiDAP), lithium has the highest positive Δ*f* value, thus targeting an F whose Δ*f* value is negative in PTFE. Thus, the reactivity of LiEDA, LiDETA, and LiDAP was tested on PTFE, polyvinylidene fluoride (PVDF), and Nafion. Based on these results, we can propose the following mechanism (Scheme 1):

### 2.3. Surface and Spectroscopic Characterizations

**Figure 2:**
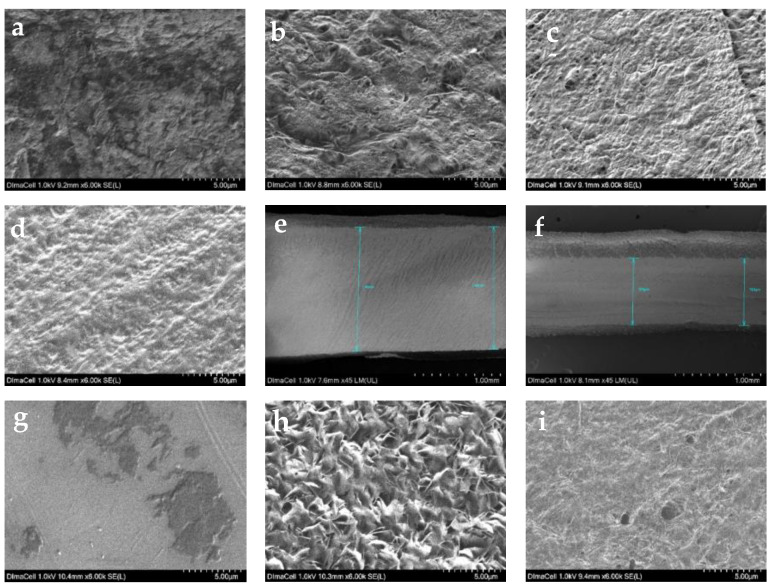
SEM images of (**a**) a PTFE surface modified by (**b**) LiEDA, (**c**) LiDETA, (**d**) LiDAP. (**e**) PTFE cross-sections (**f**) modified by LiEDA. (**g**) Pristine Nafion modified by (**h**) LiEDA and (**i**) LiDETA.

Depending on the reaction time between the alkylamide and PTFE, PVDF, or Nafion, the fluoropolymer can be modified either on the surface or in depth (Figure 2a–i). As evidenced by scanning electronic microscopy (SEM), after 6 h, the attack of PTFE is more visible on its cross-section (darker grey area in Figure 2f) compared to the pristine one (Figure 2e). For this purpose, the sample was cut after being frozen in liquid nitrogen to access to a clean slice and observe the width attacked by the chemical modification. The untreated width of pristine PTFE (Figure 2a) is about 1.4 ± 0.1 mm on average, and that of the upper treated part is 319 ± 1 µm. The width of the sample is originally 2 mm; we can assume that the cutting, even under liquid nitrogen, has flattened the material, as the total width of the sample is 1.6 ± 0.1 mm on this image. The chemical treatment has reduced the thickness. This gives us a more precise description of how the sample is affected by the treatment and how the latter operates.

The X-ray photoelectron spectroscopy (XPS) measurements on a PTFE surface modified by alkylamides clearly show a decrease in the atomic % of fluorine bound to carbon after 6 h, with a carbon-to-fluorine ratio varying from 1.2 to 0.28, respectively (Table 2). Pristine PTFE samples contain C and F atoms in a 1:2 ratio in the form of -CF_2_- bonds, while this ratio is about 7:2 for modified PTFE.

**Figure 3:**
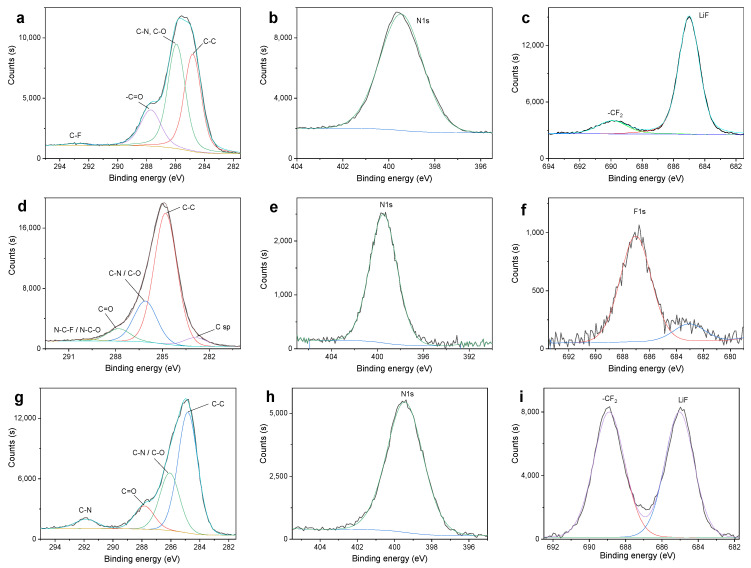
Surface analysis by XPS. XPS spectra of PTFE modified by LiEDA for (**a**) C1s, (**b**) N1s, and (**c**) F1s. XPS spectra of PVDF modified by LiDETA for (**d**) C1s, (**e**) N1s, and (**f**) F1s. XPS spectra of Nafion modified by LiEDA for (**g**) C1s, (**h**) N1s, and (**i**) F1s.

The strong signals of -CF_2_- and -CF_3_ from pristine PTFE between 292 and 294 eV disappear after LiEDA etching (Figure 3a–c) [45]. This is the same trend for PVDF etched by LiEDA (Figure 3d–f), while the peak is damped for Nafion etched by LiEDA or LiDETA (Figure 3g–i). Two configurations are possible at 286 eV: C-N and C-O; the N1s peak at 399.5 eV corresponds to C-N (Figure 3h) [46].

**Figure 4:**
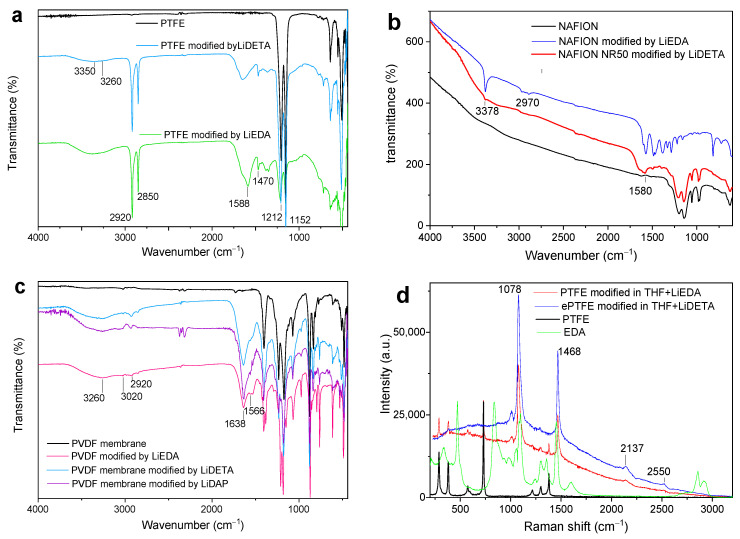
Vibrational analysis by IR-ATR and Raman spectroscopies of PTFE, PVDF, and Nafion modified by lithium alkylamides. (**a**) IR-ATR spectra of pristine PTFE and its chemical modifications by LiEDA and LiDETA. (**b**) IR-ATR spectra of pristine Nafion and its chemical modifications by LiEDA and LiDETA. (**c**) IR-ATR spectra of pristine PVDF membrane and its chemical modifications by LiEDA, LiDETA, and LiDAP. (**d**) Raman spectra of pristine PTFE and PTFE and its chemical modifications by LiEDA and LiDETA as well as EDA solvent (excitation 785 nm).

The IR attenuated total reflectance (IR-ATR) and Raman spectroscopies evidence shows clear differences between the modified fluoropolymers and their pristine form with the presence of new bands (Figure 4): -NH_2_ (around 3300 cm^−1^), -CH_2_ (2920, 2850, and 1470 cm^−1^), and -NH (1588 cm^−1^, visible for LiEDA, Figure 4a). The -CF_2_ bands of pristine PTFE fade after etching or disappear (Figure 4d), as also observed by Raman spectroscopy. These bands can be related to the monofluorinated aliphatic group, -CH_2_- alkane, vinyl CH stretching, substituted alkyne -C≡C-H stretching (2137 cm^−1^), or, probably, reactions with THF (2550 cm^−1^) and N-H stretching [47]. The modification of Nafion leads to the presence of amino groups (3377 and 1572 cm^−1^ in the IR-ATR mode) on its surface without removing the sulfo group (727, 804, 972, and 1060 cm^−1^, Figure 4b), making modified Nafion a zwitterionic membrane. The same conclusions drawn from PTFE and Nafion can be made for PVDF (Figure 4c). The presence of -CH_2_ (in the range 3020–2920 cm^−1^), -NH_2_ (around 3250 cm^−1^), and -NH- (~1560 cm^−1^) bands is due to defluorination and amination by lithium alkylamides in their respective synthesis solvent. The sulfonic group is more visible by IR-ATR (S=O stretching at 1211, 1182, and 1070 cm^−1^) than by XPS.

In addition, lithium alkylamide salts synthesized from an equimolar reaction between lithium and EDA, DETA, or DAP are soluble in THF. When dissolved in this solvent, they react with fluoropolymers.

**Figure 5:**
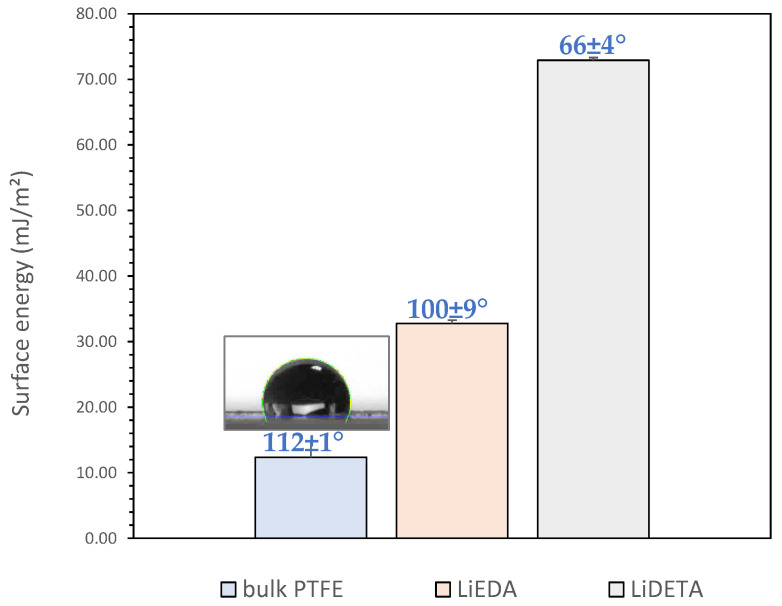
Surface free energy and corresponding contact angle values (in blue). The error bars are calculated with the propagation of uncertainty using first-order Taylor expansion applied to Fowkes’ theory formulas [11].

The presence of carbon sp2 and sp confirms the drastic rearrangement of the fluoropolymer surface (Figure 2) with the shortening of bonds. The chemical modification by lithium alkylamide produces contact angle lowering (Figure 5). This indicates a rise in the surface free energy. PTFE becomes less hydrophobic and sees its surface energy increased by three and six when modified by LiEDA and LiDETA, respectively. Furthermore, mechanical stress is then produced by the shortening of the surface bonds relative to those in the bulk, causing visible deformations. For instance, there is a contraction of the PFTE samples of approximately 14% after 6 h of treatment by LiEDA.

According to Fowkes’ theory (see Section 3.5), PTFE LiEDA appears to present a stiffer surface, with a modulus of 1.73 ± 0.30 GPa, compared to bulk PTFE, with a modulus of 1.31 ± 0.10 GPa. This latter is higher than the tensile Young’s modulus we can find in the literature (0.4 GPa). PTFE LiDETA shows no significative differences with the bulk material, with a modulus of 1.37 ± 0.08 GPa.

Contrary to Fluoroetch etchant, LiEDA PTFE presents a higher modulus than bulk PTFE, which could be explained by the shortening of the bond’s lengths, replacing C-F bonds by multiple bonds (Equation (5)). These bonds are stiffer in molecular chains, which impacts the material at a macroscale. LiDETA does not appear to have such a radical effect on the mechanical behavior of the PTFE surface, since no significant differences were observed. Thus, LiDETA seems to be a milder etchant than LiEDA in terms of mechanical properties.

### 2.4. Energy Dispersive Spectroscopy Measurements

**Figure 6:**
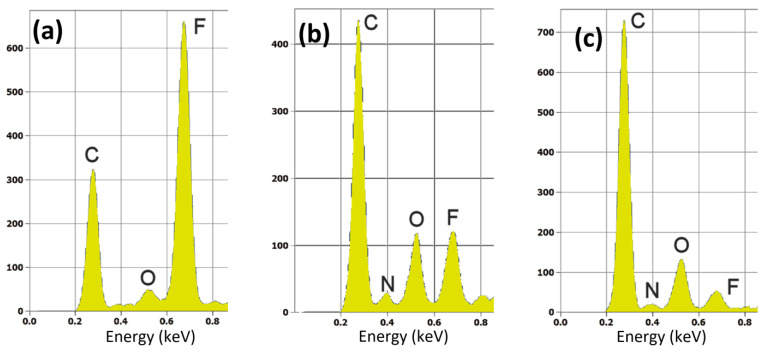
Energy dispersive spectra of (**a**) bare PTFE, (**b**) PTFE modified by LiEDA, and (**c**) PTFE modified by LiDETA.

A better understanding of the degree of amination of modified fluoropolymers by LiEDA or LiDETA comes from energy dispersive X-ray spectroscopy (EDX) measurements during SEM observations (Figure 6). Indeed, the direct identification and quantification of amino groups at an aminated surface by XPS is not possible when the amino groups coexist with a manifold of other nitrogen containing species with similar chemical shifts [48,49,50]. This is the case for nitrogen adsorbed from air on bare PTFE, even after vacuum treatment, as shown at 0.4 keV (Figure 6a), where nitrogen is expected. After the chemical treatment by LiEDA (Figure 6b) or LiDETA (Figure 6c), the fluorine peak intensity decreases drastically, especially upon LiDETA modification. The expected C/F ratio of 0.5 for bare PTFE becomes 4 for PTFE modified by LiEDA and 14 for PTFE modified by LiDETA.

The presence of oxygen at 0.5 keV on bare PTFE is due to its adsorption from air. In contrast, the oxygen peak intensity increases with LiDETA and LiEDA treatment, respectively. There is a competition between lithium alkylamides and absolute ethanol reaction during the first washing step, followed by rinsing with water. The presence of -OH groups is visible by IR-ATR at about 3400 cm^−1^ on the spectra (Figure 3).

**Figure 7:**
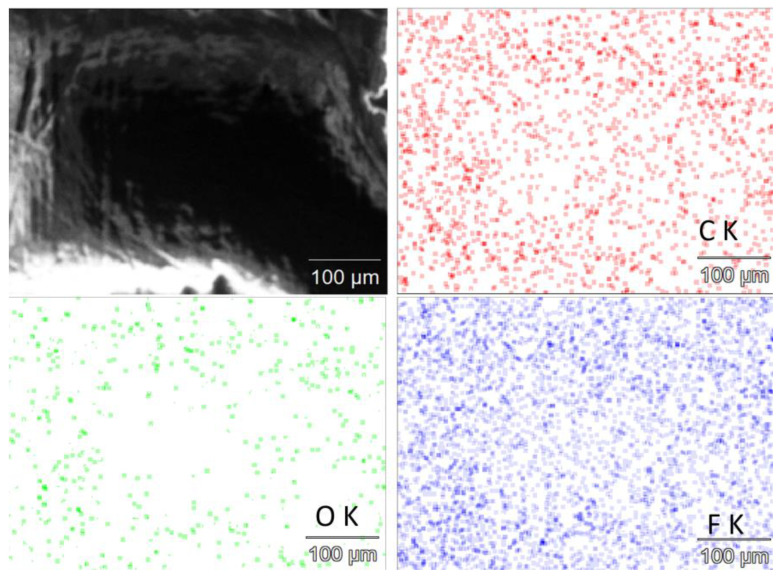
EDX mapping of C, O, and F elements of bare PTFE.

**Figure 8:**
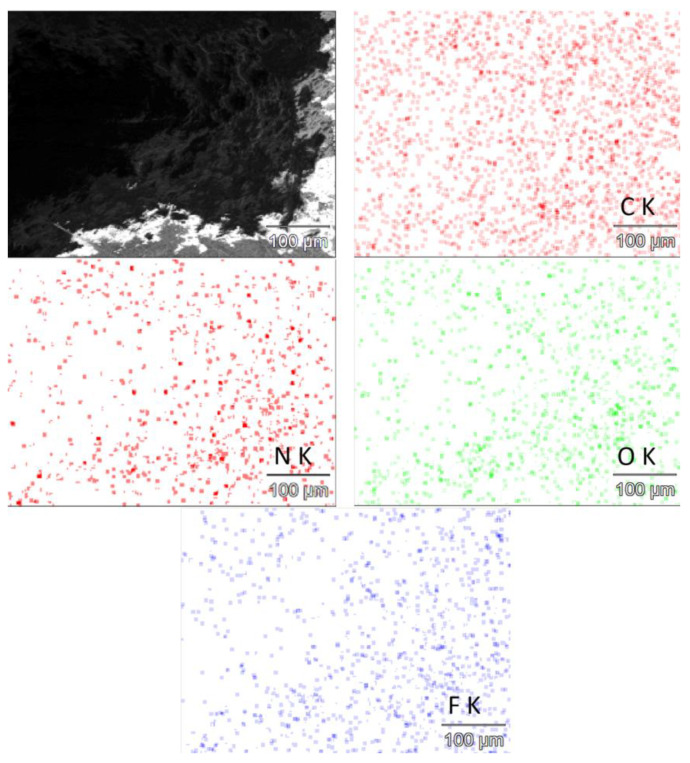
EDS mapping of C, N and O elements of the surface of PTFE modified by LiEDA.

**Figure 9:**
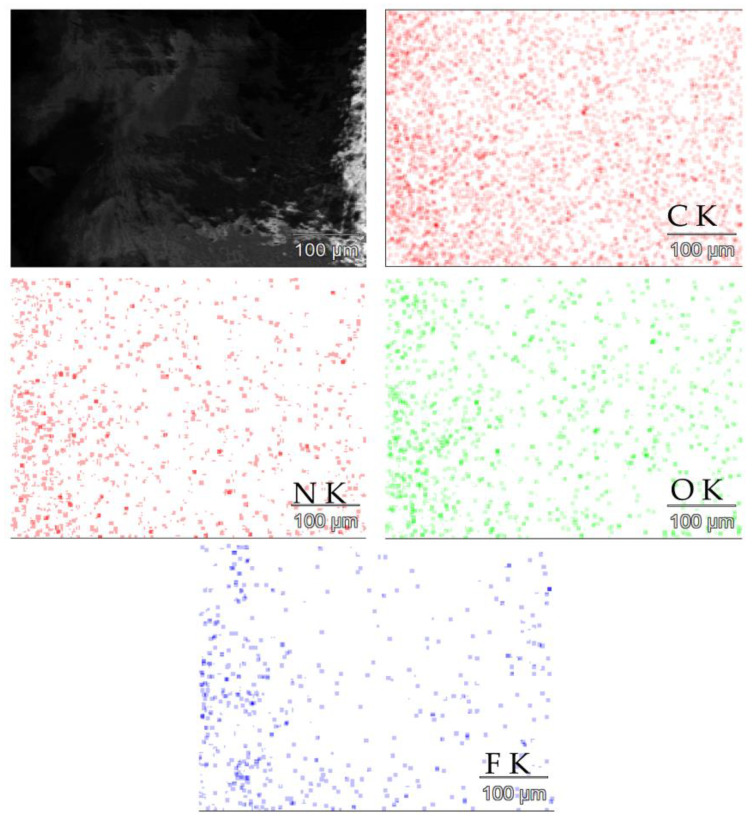
EDX mapping of C, N, O, and F elements of PTFE modified by LiDETA.

EDX mapping against the presence of elements on the surface of bare PTFE (Figure 7), PTFE modified by LiEDA (Figure 8), and PTFE modified by LiDETA (Figure 9) provides insight into their quantity and their distribution at the magnification scale of 100 µm. Globally, in Figure 7, Figure 8 and Figure 9, the elemental distributions are quite uniform.

The yield of amination is linked to the N element after chemical modifications, as listed in Table 3. This amination process also leads to alcohol functionalization during the washing and rinsing processes, as observed by IR-ATR spectroscopy but also by EDS. The yield of defluorination is linked to the F element in Table 3, with the best efficiency for LiDETA treatment.

The amination process of PTFE by lithium alkylamides, as well as the other fluoropolymers tested as a proof of concept, is a one-pot process which could be an interesting method for the remediation of PFAS. Indeed, the only byproduct is LiF, which precipitates.

## 3. Materials and Methods

### 3.1. Materials

All the chemicals are from Sigma-Aldrich (St. Quentin Fallavier, France). The PTFE and PVDF foils (2 mm thickness) are from Goodfellow (Lille, France) and of the highest purity without additives. Nafion membrane is from Sigma-Aldrich (France). All the pristine and modified surfaces were cleaned in absolute ethanol, rinsed in Millipore water under ultrasonication three times for 5 min, and dried in an oven for 1 h at 50 °C prior to the experiment in the glove box. The glove box (Jacomex, Dagneux, France) is under a permanent argon stream with oxygen and water traces under 1 ppm.

### 3.2. Experimental Conditions for the Reaction between PTFE, PVDF or Nafion, and Lithium Alkylamides

In the glove box under argon stream, a piece of 1 cm^2^ of PTFE, PVDF, or Nafion was placed in a Duran flask with a screw closure containing 10 mL of the solvent (EDA or DETA or DAP). Then, 0.2 mol of lithium is added under stirring with a glass-coated stirring rod at 1 bar and 20 °C for 6 h.

### 3.3. Analytical Methods

XPS: The surface chemical structure was analyzed by X-ray photoelectron spectroscopy using a Thermofisher Scientific Nexsa spectrometer (Waltham, MA, USA) with a monochromatic Al-Kα X-ray source (hν = 1486.6 eV, spot size = 400 µm). The use of a low-energy electron flood gun was necessary for the analysis. The photoelectron detection was carried out perpendicularly to the sample surface using a constant energy analyzer mode (pass energy 20 eV), and spectra were recorded with a 0.1 eV energy step size. Binding energies were referenced to the hydrocarbon (C–C and/or C–H) C1s peak set at 284.8 eV. Quantification was performed based on the photopeak areas after a Shirley type background subtraction using the Thermofisher Scientific Avantage© software (v6)and its “ALTHERMO1” library for sensitivity factor collection.

IR-ATR: The IR-ATR characterizations were performed on the ATR 4X module of the Jasco FT-IR 4X spectrophotometer (Tokyo, Japan) equipped with a Ge crystal (32 scans).

Raman: The Raman analyses were performed using the BWTEK confocal microRaman spectrometer (Plainsboro, NJ, USA) equipped with a high-quantum-efficiency CCD array and deep cooling (−25 °C) for high dynamic range detection using a ×20 objective lens. The acquisitions were carried out in the backscattered direction, with an integration time of 30 s. Three spectra were collected at each location and averaged to reduce the noise level using an excitation source of a 785 nm wavelength and 10% of the 500 mW output power.

Microscopy: Scanning electronic microscope (SEM) imaging was carried out by a Hitachi SU 8320 (Tokyo, Japan), and the samples were coated with approximately 6 nm of carbon using a Quorum Q150T S Plus vacuum evaporator (QuorumTech, Lewes, UK).

Contact angle: The contact angles were measured in air using an Easy Drop DSA100 (KRUSS, Hamburg, Germany), and the images were acquired using the Drop Shape Analysis software (v1.91.0.2). The measurements were performed at room temperature with a series of ten deionized water (W) and diiodomethane (DIM) drops of 10 μL as testing liquids to evaluate contact angles. These angles were used to calculate the total surface energy ($\sigma_{s}$), by the determination of its polar ($\sigma_{S}^{P}$) and dispersive ($\sigma_{S}^{D}$) terms, according to Fowkes’ theory [51].

XRD: Diffraction data were collected on a Nonius KappaCCD diffractometer equipped with a nitrogen stream low-temperature system (Oxford Cryosystems, Oxford, UK). The X-ray source was a graphite-monochromated Mo-Kα radiation (λ = 0.71073 Å) in a sealed tube.

### 3.4. Ab Initio Calculations

The local reactivity descriptors *f*^+^, *f*^−^, and *f^o^* (Fukui indices) related to nucleophilic, electrophilic, and radical attacks were calculated following the condensed dual descriptor Δ*f* to reveal reactive sites at a glance [37,38]. They were computed at the B3LYP/6-31G** level of theory and post-processed with Multiwfn 3.7 [35,36,52].

### 3.5. Contact Angle Measurements

The contact angles were measured in air using an Easy Drop DSA100 (KRUSS, Germany), and the images were acquired using the Drop Shape Analysis software. The measurements were performed at room temperature with a series of ten deionized water (W) and diiodomethane (DIM) drops of 10 μL as testing liquids in order to evaluate contact angles. These angles were used to calculate the total surface energy ($\sigma_{s}$), by the determination of its polar ($\sigma_{S}^{P}$) and dispersive ($\sigma_{S}^{D}$) terms, according to Fowkes’ theory [51]. Fowkes’ theory is the most widely used method for determining surface energy, as it uses only two liquids, contrary to Owens/Wendt theory, which requires the choice of multiple liquids. This makes it less easy to compare results for the latter method. Fowkes’s theory is based on a two-component model, which supposes that the surface energy is the result of the addition of its dispersive and polar component. A more recent method called Owens, Wendt, Rabel, and Kaelbel (OWRK) is based on Fowkes’s theory and would also be suitable for this study. The use of more than two liquids is recommended for the linear fit though.

The method and the theory presented here are extracted from the well-described KRUSS technical note TN306e. Fowkes’ theory is based on three fundamental equations that describe interactions between solids and liquids. First, there is Young’s equation:

σ*_S_* = σ*_SL_* + σ*_L_*
*cosθ* (1)

wherein σ*_L_* = the overall surface tension of the wetting liquid,

σ*_S_* = the overall surface energy of the solid, σ*_SL_* = the interfacial tension between the solid and the liquid, and *θ* is the contact angle between the liquid and the solid.

From Dupre’s definition of adhesion energy, the following is obtained:

*I_SL_* = σ*_S_* + σ*_L_* − σ*_SL_* (2)

wherein *I_SL_* = the energy of adhesion per unit area between a liquid and a solid surface.

Fowkes’ theory assumes that the adhesive energy between a solid and a liquid can be interpreted as interactions between the dispersive components of the two phases and interactions between the non-dispersive (polar) components of the two phases.

(3) $$I_{SL}=2\left[{\left(\sigma_{L}^{D}\right)}^{1/2}{\left(\sigma_{S}^{D}\right)}^{1/2}+{\left(\sigma_{L}^{P}\right)}^{1/2}{\left(\sigma_{S}^{P}\right)}^{1/2}\right]$$

wherein σ*_L_^D^* = the dispersive component of the surface tension of the wetting liquid, σ*_L_^P^* = the polar component of the surface tension of the wetting liquid, σ*_S_^D^* = the dispersive component of the surface energy of the solid, and σ*_S_^P^* = the polar component of the surface energy of the solid.

These equations combined lead to the primary equation of the Fowkes’ surface energy theory:

(4) $${\left(\sigma_{L}^{D}\right)}^{1/2}{\left(\sigma_{S}^{D}\right)}^{1/2}+{\left(\sigma_{L}^{P}\right)}^{1/2}{\left(\sigma_{S}^{P}\right)}^{1/2}=\frac{\sigma_{L}\left(cos\theta +1\right)}{2}$$

From this latter equation, a two-step determination is used. First, the component $\sigma_{s}^{D}$ is determined with a non-polar solvent, the one most used and used here is diiodomethane. It is assumed that $\sigma_{L}^{P}=0$ and $\sigma_{L}=\sigma_{L}^{D}$. The Equation (4) becomes

(5) $$\sigma_{S}^{D}=\frac{\sigma_{L}\left(cos\theta +1\right)²}{4}$$

with the $\sigma_{L}$ value taken from the Kruss DSA database and *θ* obtained by the experiment.

The second step defines the polar component $\sigma_{s}^{P}$ using water as the testing liquid. The Equation (4) becomes

(6) $$\sigma_{s}^{P}=\frac{\sigma_{L}^{2}{\left(cos\theta +1\right)}^{2}-4\sigma_{L}^{D}\sigma_{S}^{D}}{4\sigma_{L}^{P}}$$

with $\sigma_{L}$, $\sigma_{L}^{D}{,\;and\;\sigma }_{L}^{P}$ known from the Kruss DSA database and $\sigma_{S}^{D}$ calculated before. θ is obtained experimentally.

The surface energies obtained in Figure 5 describe the change in the bulk material into less hydrophobic surfaces regarding the changes caused by LiEDA and that of hydrophilic surfaces caused by LiDETA. Indeed, bulk PTFE sees its surface energy increased by three for LiEDA and six for LiDETA.

## 4. Conclusions

In summary, the direct defluorination and amination of PTFE and, by extension, fluoropolymer surfaces can be achieved simply and quickly as a one-pot synthesis under mild conditions (20 °C, 1 bar) and an inert atmosphere (argon). The chemical process involves dipping the fluoropolymer into a solution containing excess aliphatic di or triamine (EDA, EDTA, or DAP) and lithium amide formed between the lithium and the aliphatic di or triamine. Only a few hours are needed for surface modification. With prolonged contact, the fluoropolymer bulk is attacked.

In stoichiometric conditions, both lithium and amine are consumed to form a solid lithium alkylamide. When dissolved in THF, for instance, it leads to the irreversible chemical etching of fluoropolymers such as PTFE, PVDF, or Nafion.

The defluorinative amination of the C-F bond is evidenced by spectroscopic analysis such as XPS, IR-ATR, and microscopy (SEM). In contrast, the washing step with absolute ethanol followed by water rinsing introduces the presence of -OH groups on the surface of the modified fluoropolymers. This competes with amine functionalization.

Nevertheless, this reductive amination process can be regarded as a generalization of the Billups–Birch and Benkeser reductions. It paves the way for many applications based on fluoropolymers, but fluorinated compounds are concerned, while giving them a new chemical impulse. All this work can be transposed to sodium alkylamide salts, thus drastically reducing their cost.

## Figures and Tables

**Table 1:** Condensed Fukui functions and condensed dual descriptors. Condensed Fukui function f and condensed dual descriptor Δ*f* calculations based on Hirshfeld charges of LiEDA, LiDETA, LiDAP, and PTFE (relaxed separately) in a gas phase for lithium salts and in a solid phase (crystal cell) for PTFE at the B3LYP/6-31G** level of theory. The units used are elementary charge e.

	*Atom*	*f^+^*	*f^−^*	*f^o^*	Δ*f*
LiDETA 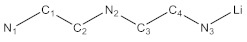	C_4_	0.0432	0.0068	0.025	−0.0365
	C_3_	0.0326	0.0045	0.0186	−0.0281
	N_2_	0.0582	0.0028	0.0305	−0.0554
	N_3_	0.2706	−0.0054	0.1326	−0.276
	Li	0.2039	0.9015	0.5527	0.6975
	C_2_	0.0109	0.002	0.0064	−0.0089
	C_1_	0.0082	0.0026	0.0054	−0.0057
	N_1_	0.0225	0.0036	0.0131	−0.0189
LiDAP 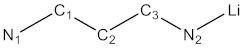	C_3_	0.0434	0.0069	0.0251	−0.0365
	C_2_	0.0288	0.0042	0.0165	−0.0246
	N_2_	0.3062	−0.005	0.1506	−0.3112
	Li	0.2165	0.9029	0.5597	0.6863
	C_1_	0.0206	0.0049	0.0128	−0.0157
	N_1_	0.0516	0.0062	0.0289	−0.0454
LiEDA 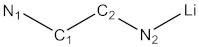	C_2_	0.048	0.0071	0.0276	−0.0408
	C_1_	0.0362	0.0058	0.021	−0.0304
	N_1_	0.0491	0.009	0.029	−0.0402
	N_2_	0.3213	−0.005	0.1582	−0.3263
	Li	0.2202	0.9042	0.5622	0.684
PTFE 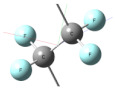	C	0.2567	0.2269	0.2418	0.0297
	F	0.1216	0.1365	0.1291	−0.0149

**Table 2:** Chemical composition (atomic %) of the surface, estimated based on XPS survey spectra.

Sample	Carbon	Fluorine	Lithium	Nitrogen	Oxygen
PTFE	35.3	64.5	-	0.1	0.1
PTFE LiEDA	36.6	30.8	18.8	7.7	5.3
PTFE LiDETA	75.1	6.0	0.5	4.1	13.8
PVDF	54.2	40.8	-	0.1	4.8
PVDF LiEDA	71.2	2.5	0.5	12.8	12.2
PVDF LiDETA	84.0	1.4	0.1	7.5	6.9
Nafion	31.9²	60.0	-	0.1	7.5
Nafion LiEDA	20.3	13.0	12.4	6.0	4.8
Nafion LiDETA	60.6	14.0	0.9	13.2	8.5

**Table 3:** Elemental analyses of the surface of bare PTFE, PTFE modified by LiEDA, and PTFE modified by LiDETA.

	PTFE		LiEDA-Modified PTFE		LiDETA-Modified PTFE	
Element	Weight %	Atom %	Weight %	Atom %	Weight %	Atom %
C K	17.2 ± 0.3	24.5 ± 0.4	23.2 ±0.4	29.5 ± 0.5	33.5 ± 0.4	40.3 ± 0.5
N k	-	-	15.2 ± 1.7	16.5 ± 1.9	16.1 ± 2.0	16.6 ± 2.1
O K	5.4 ± 0.8	5.8 ± 0.8	30.2 ± 2.4	28.8 ± 2.3	33.6 ± 2.2	30.4 ± 2.0
F K	77.4 ± 1.5	69.7 ± 1.3	31.4 ± 2.1	25.2 ± 1.7	16.8 ± 1.7	12.8 ± 1.3

## Data Availability

Data are contained within the article.
